# Testing the Recommendations of the Washington State Nutrition and Physical Activity Plan: The Moses Lake Case Study

**Published:** 2006-03-15

**Authors:** Donna B Johnson, Lynne T Smith

**Affiliations:** Center for Public Health Nutrition and Nutritional Sciences Program, University of Washington; Center for Public Health Nutrition, University of Washington, Seattle, Wash.

## Abstract

**Background:**

The Washington State Nutrition and Physical Activity Plan provides a framework in which policy makers can work together to build and support healthy environments for nutrition and physical activity. The city of Moses Lake, Wash, was chosen to serve as a pilot site to test the conceptual approaches and recommendations of the plan and to develop a model for healthy communities elsewhere in the state.

**Context:**

Moses Lake is an ethnically diverse, geographically isolated town with a population of about 15,000.

**Methods:**

An advisory committee used data from an inventory of local policies and environments, along with the recommendations from the state plan, to develop a plan for *Healthy Communities Moses Lake*. Three initiatives were chosen for the first actions: a connected system of trails and paths, enhanced facilities for breastfeeding in the community, and a community garden.

**Consequences:**

Records of cumulative actions demonstrated that *Healthy Communities Moses Lake* continued to be an active and productive project. Initial measures of success were collected by each of the three first action teams. Environmental changes will be monitored by comparison with the initial inventory of local policies. Long-term health outcomes in Moses Lake will be monitored by the Washington State Department of Health.

**Interpretation:**

*Healthy Communities Moses Lake* was successful because the city had leaders and volunteers who were committed to making the city a healthier place. Lessons learned about community-based planning and evaluation are now being applied to *Healthy Communities* initiatives.

## Background

The United States has experienced a steady increase in obesity and overweight due to changes in the environment and lifestyle behaviors that have resulted in energy imbalances for the majority of adults. The causes of this energy imbalance are complex, and solutions must be comprehensive and multisectoral (1). In 2001 and 2002, a work group composed of representatives from the following areas developed the Washington State Nutrition and Physical Activity Plan: transportation, parks and recreation, education, industry, health care, food assistance programs, advocacy groups for active transportation, food security, environmental health, and physical activity (2). The purpose of the state plan is to provide a framework in which policy makers at the institutional, community, county, and state levels can work together to build and support environments that make it easier for Washington State residents to choose healthy foods and to be physically active. The plan includes six objectives and 15 evidence-based recommendations (Table 1).

To test the conceptual approaches and recommendations of the plan and to develop a model for communities across the state, the Washington State Department of Health conducted interviews in five small, geographically isolated cities and asked government leaders to write letters if they were interested in serving as a pilot site. The city of Moses Lake was chosen as the first *Healthy Communities* site, based on its demographics and readiness to make environmental changes.

## Context

Moses Lake is located in the Columbia Basin in central Washington State. Although the economic base of the city continues to be agricultural, the city also benefits from light industry and tourism. The city has a population of about 15,000, and the surrounding unincorporated area has an additional 14,000 people. Eighty percent of Moses Lake residents identify themselves as white, 2% as black or African American, 1% as American Indian or Alaska Native, 1% as Asian, and 3% as two or more races (3). Twenty-six percent identify themselves as Hispanic or Latino (3). In 2003, the unemployment rate was 9.6% (3). There are almost 7000 children enrolled in the Moses Lake School District; 54% are enrolled in the free and reduced-price lunch program (4). Moses Lake is in Grant County, which has a population of about 75,000. Table 2 provides Behaviorial Risk Factor Surveillance System (BRFSS) data for nutrition and physical activity behaviors and related health outcomes in Grant County and Washington State.

## Methods

A timeline for the *Healthy Communities Moses Lake* plan through 2002 is presented in Table 3. The city of Moses Lake, the Moses Lake Business Association, the Grant County Public Health District, the National Park Service's (NPS's) Rivers, Trails, and Conservation Assistance Program, the Washington State Department of Health (DOH), and the University of Washington (UW) Center for Public Health Nutrition and Health Promotion Research Center provided initial leadership. Fifty-two Moses Lake leaders and residents came to the first meeting of the *Healthy Communities* advisory committee. Table 4 lists the agencies represented on the advisory committee. The first responsibilities of the advisory committee were to participate in an inventory and planning process, ensure representation of community interest groups in the planning process, and participate in a kick-off walk and health fair in July and a ceremonial presentation of the plan to the community in November.

A team of *Healthy Communities Moses Lake* volunteers completed an inventory of existing institutional, community, and municipal policies and environments in Moses Lake. The inventory tools were designed to collect information for each of the six nutrition and physical activity objectives of the state plan. Inventory results were presented to the advisory committee in August 2002 (5).

In September, an ad hoc work group composed of advisory committee members met to develop policies and actions for *Healthy Communities Moses Lake*. The meeting was facilitated by NPS staff. The group developed a vision statement: "Residents of the Moses Lake area enjoy an active, healthy lifestyle that includes nutritious foods, recreation, and positive interactions with each other." A SWOT (strengths, weaknesses, opportunities, and threats) analysis of the vision statement was performed. After reviewing the objectives and recommendations made in the state plan, the work group discussed and developed a list of possible strategies and actions. Through a voting process, the group selected three projects for the first year of the plan: 1) a connected, attractive, and safe path and trail system; 2) enhanced facilities for breastfeeding in the community; and 3) community gardens. The work group presented its action plan to the advisory committee 1 week later, and it was approved. A team was established for each of the three projects to develop short- and long-term goals and a timeline for meeting these goals. Since then, each of the three teams has met at least monthly, and the advisory committee has met twice annually.

During fall 2002, the three teams and advisory committee leaders wrote *Healthy Communities Moses Lake: An Action Plan to Promote Nutrition and Physical Activity* (6) with technical assistance from NPS, UW, and DOH staff. The plan included the vision statement and work plans for each of the three initial projects. The plan was presented to the community in a ceremony at the last farmers' market of the year in November and approved by the Moses Lake City Council and Grant County Public Health District in December.

A local leadership structure was established in early 2004 to develop a sense of responsibility for sustaining the program. Before then, most meetings of the advisory committee were facilitated by DOH staff. The core leadership group now develops agendas, facilitates meetings, recruits members, and promotes outreach and integration with other community efforts. The group includes one leader from each of the projects and one representative each from Moses Lake and the Grant County Public Health District, and a part-time staff member of the Moses Lake Business Bureau coordinates the group's activities.

Evaluation plans for *Healthy Communities Moses Lake* included the following: 

- Compilation of cumulative actions abstracted by UW evaluators from meeting minutes, progress reports, survey and evaluation reports, newspaper clippings, event flyers, and grant applications (7)
- Qualitative data on the process of community organization collected through telephone surveys by UW evaluators (8)
- Measures of success as defined and collected by the trails and paths, breastfeeding, and community gardens teams
- Monitoring by DOH of long-term population changes in behavior and health outcomes by comparing BRFSS data from an oversample in 2003 with future oversamples
- Detecting environmental changes by comparing the policy inventory completed by volunteers at the beginning of the project to future inventories

## Consequences

Table 5 provides information about cumulative actions taken as part of *Healthy Communities Moses Lake.* In August 2004, a random-digit–dial telephone survey of 350 (313 English-speaking, 37 Spanish-speaking) adults was conducted to assess the results of a diabetes awareness campaign in Moses Lake. In addition to questions designed to measure the effect of the diabetes awareness campaign, respondents were asked six questions about *Healthy Communities Moses Lake*; their answers are presented in Table 6. Actions taken by each of the community teams are highlighted in the following sections and outlined in Table 7.

### The trails and paths team

The goal of the trails and paths team was to increase the use of a network of linked walking and biking trails throughout the city. An initial assessment found that the existing trails were underused, either because many people did not know about them or because the trails did not lead to popular destinations. In 2003, initial successes included improvements in signage; safety and amenities such as water facilities, bike racks, benches, restrooms, and lighting for 26 miles of existing trails; and distribution of a trail map.

In October 2003, NPS staff led a trail-planning *charette* — an intensive effort to develop conceptual plans within compressed, creative, high-energy sessions. The charette included community volunteers and representatives from the Bicycle Alliance of Washington, Audubon Society, Washington State Department of Transportation, and Washington Chapter of the American Society of Landscape Architects. In addition to the components of a workshop, a charette involves production of plans and concepts based on the input of all participating interests. During the Moses Lake charette, volunteer design professionals quickly grasped local problems and devised solutions with drawings, working with local community members, leaders, and other technical experts.

In preparation for the charette, an assessment questionnaire was mailed to 13,000 households in the Moses Lake ZIP code area. The purpose of the questionnaire was to gather information about local needs and opinions and to inform the public about the trail-planning effort. The questionnaire was written in English and Spanish. Volunteers from the trails and paths team arranged and promoted return boxes in local grocery stores and at city offices. People who returned the questionnaires were entered into a drawing for passes to a local tourist attraction. The questionnaire was returned by 727 residents. Forty-three percent of respondents indicated that they knew nothing or very little about the existing walking and bike trail system, 34% of respondents indicated that they were willing to help with the planning efforts, and 63% of respondents indicated that they would use an improved trail system daily or weekly. The most commonly requested improvements, in order of importance, were the following: 1) separation from traffic, 2) safety and security, 3) good lighting, 4) access to toilets, 5) landscaping or scenic views, 6) benches, and 7) access to drinking water. The trails and paths team thought that the responses reflected their own experience as residents as well as results from focus groups that had been conducted with representative samples of Moses Lake residents.

The charette produced a master plan for an integrated trail system. The plan was published in March 2004 as a special supplement in the local newspaper and distributed at several community events. The trails plan has been adopted by the Moses Lake City Council as part of the Parks and Recreation Comprehensive Plan and by Grant County as part of the 6-year road plan. There are now several projects in the design and funding stages that will result in 10 or more miles of new trails and connections between existing trails. Since the work of the team began, civic groups have volunteered to improve sections of existing trails, businesses have donated land to the trails system, and several city and county regulations and ordinances have changed to include trail development as part of transportation construction and renovation projects.

A laser counting system (PTC-3 Passive Trail Counter, Carson Electronics, Valemount, British Columbia) was purchased to obtain baseline and follow-up information on trail use. The goal of the trails and paths team was to take annual measurements of trail use in both Moses Lake and a control community in Grant County through at least 2008. Data were collected at nine sites on the trails in Moses Lake for a week in spring 2003 and again in 2004. Mean daily trail use was 182 individuals in 2003 and 191 individuals in 2004, with a mean (SD) increase in trail use of 8.7 (6.2) individuals per day. Data for the control community were collected in 2003 but were not collected in 2004 because of confusion between city staff in Moses Lake and staff in the control community.

### The Moses Lake Breastfeeding Coalition

Breastfeeding team members formally organized as the Moses Lake Breastfeeding Coalition. In 2003 and 2004, the 62 members of the coalition developed a Web site (www.mlbreastfeeding.org/), trained licensed child care providers, hosted a luncheon for human resources staff to introduce them to the need for worksite policies that support breastfeeding, provided breastfeeding equipment for seven worksites, provided awards for employers who actively support employees who breastfeed, and established nursing rooms at local businesses and the Grant County Fair. To increase knowledge and acceptance of breastfeeding, the coalition also provided training and resources for health care providers and staff; worked with local news media, educators, and civic groups; and sponsored a short trailer in all six theaters at a local movie complex for 1 year. The coalition increased the attendance of representatives from health care, child care, and worksite settings at coalition meetings and training sessions.

There is only one hospital in Moses Lake, and staff and administrative turnover has made progress toward changing hospital policies on breastfeeding difficult. The hospital has accepted help from the breastfeeding coalition in the form of a breastfeeding chair, a breast pump, written materials for mothers, and technical support for breastfeeding mothers.

### The community garden team

The community garden team developed land next to City Hall into a series of raised garden beds and first made the plots available to community residents in spring 2003. The garden team also provided a tool shed, free soil amendments, shared tools, and a watering system. Master gardeners taught classes and provided consultation, and local businesses and individuals donated seeds, equipment, and labor. The goals of the project were to encourage residents to gather, garden, and grow healthy food; enjoy healthy leisure activities; learn about gardening, nutrition, and preparing food; and eat more fruits and vegetables. All 61 plots were reserved within the first year. In 2004, 10 new plots, a state-of-the-art composting system, and a greenhouse were added; benches and paths were enhanced.

Twenty-nine of 61 gardeners completed evaluation surveys in fall 2003. Of those 29, 21 reserved a plot for the following year. More than half of the survey respondents reported that they ate more fruits and vegetables while they participated in the garden, and 17 of the 21 who responded to a question about finances stated that they used the garden to stretch their food dollars. Additional benefits of the garden listed by survey respondents included building a sense of community and providing access to garden space for people who did not have suitable space where they lived. In the second season, 12 of the 71 gardeners completed a different survey that did not allow comparison between years. All 12 second-year respondents indicated that they thought that their involvement in the garden helped them to lead healthier lifestyles.

### The youth wellness team 

In 2004, a youth wellness team from the Columbia Basin Job Corps joined the community garden team. Job Corps youth helped with the main community garden and also created their own garden on the Job Corps site. As Job Corps participants learned about gardening, nutrition, and physical activity, they began to advocate for changes at the Job Corps campus. Job Corps staff report that they now serve fresh fruits and vegetables in the dining room each day and that the vending machines include more healthy choices.

## Interpretation

Moses Lake was selected as a pilot site to test the objectives and recommendations of the Washington State Nutrition and Physical Activity Plan. The plan's focus is on changing policies and environments, and it is designed to be complementary to educational efforts to change individual behaviors. Some teams found it easier than others to implement the state plan's recommendations for environmental change. For example, building a connected system of trails is clearly a way to change environments, and planning and funding for trails is driven by policy. On the other hand, breastfeeding coalitions have a long history of providing support and education to individual women and health care providers, and it was more difficult for this group to improve breastfeeding policies and environments.

Sustainability of *Healthy Communities Moses Lake* depends on institutionalizing policy and environmental changes. The initiative has resulted in local government plans and budgets for trails and community gardens that will influence nutrition and physical activity choices of Moses Lake residents for years to come. Changes to hospital policies for breastfeeding would offer similar long-lasting benefits for the families of Moses Lake.

Because this pilot project was undertaken to determine whether the recommendations of the state plan were effective in communities, evaluation was an important component for UW and DOH staff. However, community members were often more interested in the work itself. As each of the three work teams developed their initial proposals, they were also asked to include measures of success for their objectives. After 2 years, some of the original team members were no longer involved, and information about some of the measures was not being collected. The short initial evaluation training and limited ongoing technical assistance to the teams was not enough to ensure that evaluation was fully integrated into the projects. As a result, UW and DOH staff are developing more effective community-based evaluation procedures for *Healthy Communities* projects.

The state plan calls on policy makers from multiple sectors to work together to build healthy environments, but the emphasis on inclusiveness also provides challenges to maintaining a strong coalition that stays focused on a common mission over time. After the three initial projects were selected, representatives of other community interests were less inclined to continue as active participants in *Healthy Communities Moses Lake*. Work has not yet moved beyond the three initial projects to include the wider variety of venues and approaches that were included in the first plan for Moses Lake. A concerted effort and skilled leadership from the Moses Lake community will be needed to sustain a focus on the common mission and integrate *Healthy Communities* into other community improvement efforts.

*Healthy Communities Moses Lake* was possible because the city had leaders and volunteers who had a strong interest in making Moses Lake a healthier place. The city has been ready for change, has made progress over a short period, and has demonstrated that many of the ideas from the Washington State Nutrition and Physical Activity Plan can be effective in local communities.

## Figures and Tables

**Table 1:** Objectives and Recommendations of the Washington State Nutrition and Physical Activity Plan

**Objective**	**Priority Recommendations**
Increase the number of physical activity opportunities available to children	Adopt school-based curricula and policies that provide quality, daily physical education for all students Encourage policies that provide kindergarten through 12th-grade (K–12) students with opportunities for physical activity outside of formal physical education classes Provide opportunities to replace sedentary behaviors, such as watching television, with physical activity
Increase the number of people who have access to free or low-cost recreational opportunities for physical activity	Provide adequate funding for state and local recreation sites and facilities Develop model policies to increase access to public facilities for physical activity Increase the number of worksites that have policies that enhance activity opportunities
Increase the number of active community environments	Use urban planning approaches — zoning and land use — that promote physical activity Incorporate transportation policy and infrastructure changes to promote nonmotorized transit Enhance safety and perceived safety to improve community walkability and bikeability
Ensure access to health-promoting foods	Increase the consumption of vegetables and fruits Ensure that worksites provide healthful foods and beverages Ensure that K–12 schools provide healthful foods and beverages
Reduce hunger and food insecurity	Provide adequate support for nutrition and food programs Improve access to nutrition programs
Increase the proportion of mothers who breastfeed their infants and toddlers	Ensure that health care settings, child care facilities, and worksite environments are breastfeeding friendly

**Table 2:** Nutrition and Physical Activity Behaviors and Related Health Outcomes in Grant County and Washington State, 2003 Behavioral Risk Factor Surveillance System

**Health Indicator**	**Grant County**	**Washington State**

	**% of Respondents (95% CI^a^)**	**% of Respondents (95% CI^a^)**
Has ever been told by a health professional that he/she has diabetes	7.6 (5.0-11.6)	6.9 (6.2-7.1)
Has ever been told by a health professional that he/she has hypertension	26.1 (20.8-32.1)	23.8 (23.1-24.6)
Has ever been told by a health professional that he/she has high cholesterol	39.3 (32.2-46.7)	33.3 (32.4-34.3)
Is overweight (body mass index = 25.0 to 29.9)	39.7 (33.3-46.5)	36.9 (35.9-37.8)
Is obese (body mass index ≥30.0)	27.1 (21.6-33.4)	21.8 (21.0-22.6)
Consumes fruits and vegetables at least five times per day	21.0 (16.1-26.9)	23.3 (22.5-24.1)
Performs sufficient physical activity during leisure time	41.4 (35.1-48.1)	53.8 (52.8-54.7)
Performs sufficient physical activity during work and leisure time	54.7 (47.9-61.4)	62.9 (62.0-63.8)

a CI indicates confidence interval.

**Table 3:** Major Advisory Committee Planning Events and Actions for *Healthy Communities Moses Lake,* Washington State

**Date**	**Events and Actions**
April 2002	Moses Lake selected as the pilot community for implementing the Washington State Nutrition and Physical Activity Plan (state plan) objectives
May 2002	Initial meeting with Moses Lake City Council and Grant County Board of Health
	Local *Healthy Communities* coordinator hired in Moses Lake
June 2002	Advisory committee members recruited
	First advisory committee meeting: introduction of the *Healthy Communities* initiative, discussions on the nutrition and physical activity environment in Moses Lake, and recruitment of volunteers for the community inventory
July 2002	Kick-off event with mayor, Grant County health officer, and Washington State health officer
July–August 2002	Inventory subcommittee audit conducted of the nutrition and physical activity environment in Moses Lake
	Second advisory committee meeting: results of inventory and objectives and recommendations of state plan discussed
September 2002	Retreat held by planning work group to choose strategies and actions
	Third advisory committee meeting: planning work group proposal presented to advisory committee
	Project work teams formed for trails and paths, breastfeeding, and community garden
November 2002	*Healthy Communities Moses Lake: An Action Plan to Promote Nutrition and Physical Activity* introduced to community
December 2002	Plan approved by Moses Lake City Council and Grant County Board of Health

**Table 4:** Sectors Represented by the *Healthy Communities Moses Lake* Advisory Committee, Washington State

City of Moses Lake	
	City Council
	Parks and Recreation Department
	Fire Department
	Police Department
	Municipal Services
	Tourism Commission
Moses Lake Business Association	
Moses Lake School District	
Samaritan Healthcare	
Port of Moses Lake	
Grant County Commission	
Grant County Public Health District	
Fitness centers	
Moses Lake Community Health Center	
Columbia Basin *Herald*	
Columbia Basin Job Corps	
Moses Lake Food Bank	
Columbia Basin Farmers' Market & Craft Bazaar	
Senior Opportunity & Services	
Boys & Girls Club of the Columbia Basin	
Washington State University Cooperative Extension	
Hispanic Youth Services	
People for People	
Take Off Pounds Sensibly (TOPS)	

**Table 5:** Actions Taken as Part of *Healthy Communities Moses Lake*, Washington State

**Action**	**August-December 2002**	**January-June 2003**	**July-December 2003**	**January-June 2004**	**Total**
Assessment and evaluation	7	22	5	2	36
Meetings	9	27	14	23	73
Community events	1	8	11	11	31
Communication^a^	5	23	9	13	50
News media coverage	1	4	2	14	21
Training, grant writing, other actions	5	27	10	15	57
Totals	28	111	51	78	268

a Communication includes newsletters, conference presentations, publications, and reports.

**Table 6:** Results of Random-Digit–Dial Telephone Survey (n = 350), 2004, *Healthy Communities Moses Lake  *

**Question**	**Respondents Who Answered Yes, No. (%)**
Have you heard about the *Healthy Communities* *Moses Lake* projects?	100 (28.6)
Have you heard about the Moses Lake community garden?	175 (50.0)
Have you heard about the Moses Lake trails team?	65 (18.6)
Have you heard about the Moses Lake Breastfeeding Coalition?	77 (22.0)
Have you participated in any way in any of the Moses Lake projects mentioned?	21 (6.0)

**Table 7:** Major Action Steps Taken by *Healthy Communities Moses Lake* Work Groups, Washington State

**Date**	**Events and Actions**

**Trail planning team**	

April 2003 to present	City staff, volunteers, and partners begin to make improvements to trails including paving, plantings, signage and bike racks; service groups (e.g., Rotary Club) adopt trails for maintenance and clean-up
July 2003	*Healthy Communities* trails plan amended to Moses Lake city plan
October 2003	Planning charette results in comprehensive long-term plan for integrated system of trails for walking and biking
March 2004	Charette results and plans published as special supplement in the Columbia Basin *Herald* and distributed at community events
September 2004	Big Bend Community College joins paths to trail system
May 2005	Moses Lake receives $340,000 Interagency Committee on Outdoor Recreation grant for Heron Trail project

**Breastfeeding coalition**	

March 2004	Worksite representatives trained on policies to support breastfeeding mothers and infants
	Breastfeeding rooms established at worksites, community settings, and hospital
April 2004	Child care providers trained on policies to support breastfeeding mothers and infants
September 2004	Health professionals trained on policies to support breastfeeding mothers and infants

**Community garden team**	

January-June 2003	First raised beds prepared in city land next to City Hall and first gardeners recruited
May-October 2003 and 2004	Cooperative extension and master gardeners provide technical assistance and weekly news media exposure
April-June 2004 and 2005	Garden site improved

**Youth wellness team**	

Fall 2003	Youth wellness team developed at Job Corps campus
Spring 2004	Youth advocacy leads to improvements in vending-machine and cafeteria foods on campus
Spring 2005	Youth wellness team establishes garden and composting on campus
